# Construction and Evaluation of Recombinant Adenovirus Candidate Vaccines for Chikungunya Virus

**DOI:** 10.3390/v14081779

**Published:** 2022-08-15

**Authors:** Liang Cao, Wei Wang, Wenchao Sun, Jinyong Zhang, Jicheng Han, Changzhan Xie, Zhuo Ha, Yubiao Xie, He Zhang, Ningyi Jin, Huijun Lu

**Affiliations:** 1 College of Laboratory, Jilin Medical University, Jilin 132013, China; 2 Key Laboratory of Jilin Province for Zoonosis Prevention and Control, Changchun Veterinary Research Institute, Chinese Academy of Agricultural Sciences, Changchun 130117, China; 3 College of Animal Science and Technology, Guangxi University, Nanning 530000, China; 4 Institute of Virology, Wenzhou University, Wenzhou 305006, China

**Keywords:** Chikungunya virus, recombinant adenovirus vaccine, glycoproteins, immunogenicity, mice

## Abstract

Chikungunya virus (CHIKV) is a mosquito-borne virus. The emergence of CHIKV infection has raised global concern, and there is a growing need to develop safe and effective vaccines. Here, adenovirus 5 was used as the vaccine vector to construct recombinant adenoviruses expressing CHIKV E2, E1, and E2-6K-E1, respectively. And then the immunogenicity and protective efficiency against CHIKV were evaluated in BALB/c mice. Compared to the ad-wt control group, all three vaccines elicited significant humoral and cellar immune responses. The levels of neutralizing antibodies in the rAd-CHIKV-E2-6K-E1 and rAd-CHIKV-E2 groups both reached 1:256, which were 3.2 times higher than those in the rAd-CHIKV-E1 group. Furthermore, the levels of lymphocyte proliferation in rAd-CHIKV-E2-6K-E1 group were the highest. Besides, the concentrations of IFN-γ and IL-4 in mice immunized with rAd-CHIKV-E2-6K-E1 were 1.37 and 1.20 times higher than those in ad-wt immunized mice, respectively. After the challenge, mice in the rAd-CHIKV-E2-6K-E1 and rAd-CHIKV-E2 groups lost 2% of their body weight compared with 5% in the ad-wt control group. And low viral loads were detected in the heart, kidney, and blood of mice immunized with rAd-CHIKV-E2-6K-E1 and rAd-CHIKV-E2 at 3–5 dpc, which decreased by 0.4–0.7 orders of magnitude compared with the ad-wt control. Overall, these data suggest that the recombinant adenovirus is a potential candidate vaccine against CHIKV.

## 1. Introduction

Chikungunya virus (CHIKV) is a mosquito-borne single-stranded RNA virus of the *alphavirus* genus (group IV) in the *Togaviridae* family [1]. CHIKV causes Chikungunya fever (CHIKF) which show high fever, joint pain, and a macropapular rash. The virus was first detected during an epidemic in Tanzania in East Africa in 1953 [1,2,3]. Since then, the virus has evolved from being an obscure viral infection to an international threat with expanding epidemics across several continents [4]. The extensive outbreaks of CHIKF in recent years have made it a global public health problem.

The viral genome consists of a 5′ methylated terminal cap untranslated region (UTR), followed by RNA coding for four non-structural proteins (nsP1–4), five structural proteins (C–E3–E2–6K–E1), and a 3′ terminal poly-A tail [5,6]. The two envelope glycoproteins E2 and E1 assemble into spikes on the virion surface and associate as trimers of heterodimers (E2–E1) on the particle surface, and are involved in the attachment and entry of the virion into susceptible target cells during subsequent infection [7,8]. E2 is the important target of neutralizing antibodies, while E1 is mainly involved in membrane fusion [9].

Recombinant adenoviruses have been proved to be safe and valuable as vaccine vectors through extensive human trials, because they can induce T cell responses and produce neutralizing antibodies with potential immunogenicity [10]. The non-replicating adenovirus vaccine vectors contain deletions in E1 and E3 of the adenovirus 5 (Ad5) genome. These deletions, coupled with multiple engineered transgene-expression sites, allow the insertions of multiple antigens at different locations, or a large antigen inserts at selected locations within the Ad5 genome, which have been used to produce recombinant vaccines [10,11].

In this study, an adenovirus 5-vector was used to construct recombinant adenovirus vaccines expressing E2, E1, or E2-6K-E1 of CHIKV. We also aimed to verify the immunogenicity and protective efficacy of three vaccines in mice.

## 2. Materials and Methods

### 2.1. Cell Culture, Virus, and Animals

HEK293, Vero, and *Aedes albopictus* C6/36 cells were maintained in Dulbecco’s modified Eagle’s medium (Hyclone) supplemented with 10% fetal bovine serum and incubated at 37 or 28 °C under 5% CO_2_. The CHIKV 0706a TW isolation strains (accession number: FJ807897) were propagated in C6/36 cells. 4–6 weeks female BALB/c mice were purchased from Beijing Vital River Laboratory Animal Technology Co., Ltd. (Beijing, China).

### 2.2. Construction and Harvest of Recombinant Viruses

The E2, E1, E2-6K-E1 protein genes of CHIKV with *Kpn* I and *Xho* I sites were synthesized by Comate Bioscience Co., Ltd. (Changchun, China). The shuttle plasmids were co-transfected in HEK293 cells with the backbone plasmid pacad5 9.2–100 by X-tremeGene HP DNA Transfection reagent (Roche, Basel, Switzerland). The recombinant adenoviruses named rAd-CHIKV-E2, rAd-CHIKV-E1, and rAd-CHIKV-E2-6K-E1 were collected until Ad-related cytopathic effects were observed. The virus titers were measured by plaque forming units.

### 2.3. Expression of E2, E1, E2-6K-E1 Glycoprotein of CHIKV

A monolayer of HEK293 cells was infected with the positive recombinant adenovirus rAd-CHIKV-E2, rAd-CHIKV-E1, and rAd-CHIKV-E2-6k-E1 at a multiplicity of infection of 0.1, respectively. And then the cells were harvested 48 h post infection. A western blotting was performed to detect protein expression as previously described except for the mouse anti-CHIKV polyclonal antibodies (provided by Dr. Jin) [6].

### 2.4. Immunization Schedule of Recombinant Vaccines in Mice

A total of 50 female BALB/c mice were randomly and equally divided into 5 groups. Four groups of mice were intramuscularly (i.m.) immunized with 5 × 10^9^ pfu/100 μL of rAd-CHIKV-E2, rAd-CHIKV-E1, rAd-CHIKV-E2E1 or ad-wt respectively, and the last group was immunized with 100 μL of PBS as a mock control (Table 1). All groups received booster immunization, in the same way, three weeks after the primary immunization. Blood samples were collected from the intravenous iliac vein/post-balloon vein after anesthesia with an appropriate amount of sodium pentobarbital.

### 2.5. Challenge Assay of Mice

Mice were challenged with 100 μL CHIKV (10^4^ pfu) via tail vein at 35 days post immunization (dpi) to test the protection of recombinant adenovirus vaccines. The changes in body weight of the mice were then monitored for seven days post-challenge (dpc). The organs of the brain, heart, liver, spleen, lung, kidney, and blood were collected by the intravenous iliac vein/post-balloon vein after anesthesia with an appropriate amount of sodium pentobarbital at 1, 3, 5, and 7 dpc for further detection of the viral loads.

### 2.6. Quantitative RT-PCR

The total RNA of the brain, heart, liver, spleen, lung, kidney, and blood samples was extracted as previously described [12]. The primers for CHIKV were forward: 5′-ACGCAGTTGAGCGAAGCAC-3′ and reverse 5′-CTGAAGACATTGGCCCCAC-3′ [13].

### 2.7. Evaluation of Humoral Immune Responses

#### 2.7.1. Determination of Antibody Titers

The anti-CHIKV IgG response against recombinant adenoviruses was determined through indirect ELISA at 7, 14, 21, 28, and 35 days-post immunization as previously described [14].

#### 2.7.2. Detection of Neutralization Antibodies

Plaque reduction neutralization test (PRNT) was used to evaluate the neutralization activity of serum samples of mice immunized with rAd-CHIKV. Titers were expressed as the reciprocal of the highest serum dilution [15].

### 2.8. Assessment of Cellular Immune Responses

#### 2.8.1. Measurement of Cytokines

The levels of cytokines were measured in the serum collected from mice immunized at 14 dpi and 35 dpi. The levels of serum IFN-γ and IL-4 were compared among the experimental groups using commercial mouse uncoated ELISA kits (Thermo Fisher Scientific, Waltham, MA, USA).

#### 2.8.2. Lymphocytes Proliferation Assay

Splenic lymphocytes were isolated and collected from the immunized mice at 35 dpi following an appropriate dose of pentobarbital sodium as previously described [12]. The stimulation index was also used to evaluate the proliferation ability of lymphocytes.

### 2.9. Statistical Analysis

Means of measures among the different immunization groups were compared by one way ANOVA followed by pairwise comparisons by LSD-t tests using GraphPad Prism software 7.0 (San Diego, CA, USA). Data are presented as the mean ± standard deviation (SD). * *p* < 0.05, ** *p* < 0.01, *** *p* < 0.001, and **** *p* < 0.0001 indicated the significance at statistical levels of 0.05, 0.01, 0.001, and 0.0001, respectively.

## 3. Results

### 3.1. Construction and Identification of Recombinant Viruses

**Figure 1:**
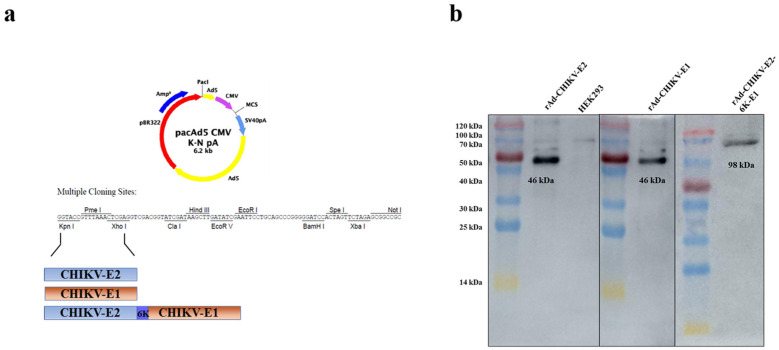
Construction and identification of the recombinant adenovirus vaccines. (**a**) The exogenous genes of E2, E1 and E2-6k-E1 of CHIKV inserted in the adenovirus 5 shuttle plasmid of pacad- shuttle CMV K-N pa vector with Kpn I and Xho I sites upstream and downstream of the open reading frame, respectively. (**b**) Identification of the protein of CHIKV E2, E1 and E2-6K-E1 proteins expressed in an adenovirus 5 vector by Western blotting.

The E1/E3 deleted replication-defective Ad5 recombinant viruses encoding full-length E2/E1/E2-6K-E1 gene were harvested when the cells’ cytopathic effects were observed. Western blot analysis showed that the recombinant protein was effectively expressed at the expected size after infecting HEK293 cells with rAd-CHIKV-E2, rAd-CHIKV-E1, and rAd-CHIKV-E2-6K-E1(Figure 1b).

### 3.2. CHIKV Recombinant Adenovirus Elicits Higher Humoral Immune Responses

**Figure 2:**
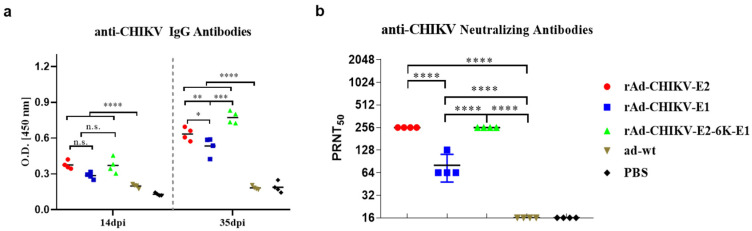
Characterization of anti-CHIKV humoral immunity response induced by the recombinant adenovirus viruses. (**a**) Anti-CHIKV-specific IgG antibody levels in the mouse serum (n = 4) were measured at 14 and 35 dpi using an ELISA. (**b**) Neutralizing antibody titer (n = 4) at 35 dpi measured by determining the reciprocal of the highest serum dilution producing a 50% reduction in PRNT_50_. Data represent the mean ± SD of each group. * *p* < 0.05, ** *p* < 0.01, *** *p* < 0.001, **** *p* < 0.0001, n.s. no significance.

To assess the immunogenicity of the CHIKV recombinant adenovirus candidate vaccines, the anti-CHIKV specific antibodies in the serum samples were detected by ELISA at 14 and 35 days post-prime immunization. It was found that rAd-CHIKV-E2-6K-E1 induced stronger CHIKV specific antibody responses than rAd-CHIKV-E2 or rAd-CHIKIV-E1 at 35 dpi (*p* < 0.01 or *p* < 0.001, respectively), but there was no significant difference observed at 14 dpi (Figure 2a). Furthermore, the CHIKV specific antibody titers induced by rAd-CHIKV-E2 were significantly higher than those induced by rAd-CHIKV-E1 at 35 dpi (*p* < 0.05). Similarly, no significant difference was observed at 14 dpi between the two above groups. In order to verify whether the cytokines were stimulated by recombinant adenovirus, mouse serum at 7 dpc (42 dpi) was collected to test the cytokines. The results showed that mice immunized with rAd-CHIKV-E2-6K-E1 and rAd-CHIKV-E2 were significantly higher than the ad-wt control group (rAd-CHIKV-E2-6K-E1, *p* < 0.001; rAd-CHIKV-E2, *p* < 0.05) in IFN-γ, as well as in IL-4 (rAd-CHIKV-E2-6K-E1, *p* < 0.001; rAd-CHIKV-E2, *p* < 0.01). The levels of IFN-γ and IL-4 in mice immunized with rAd-CHIKV-E2-6K-E1 were 1.45 and 1.38 times higher than those in the ad-wt control group, respectively. The levels of anti-CHIKV specific antibodies in the immunization groups were significantly higher than those in the control group at 14 and 35 dpi (*p* < 0.0001).

Neutralizing antibodies play an important role in CHIKV infection and viral clearance. Compared with the control group, mice of all immunization groups could produce neutralizing antibodies after booster immunization. Low levels of neutralizing antibodies in the rAd-CHIKV-E1 group were detected (1:80). Comparatively, both rAd-CHIKV-E2 and rAd-CHIKV-E2-6K-E1 could induce higher neutralizing antibody titers (1:256), which was 3.2 times than the rAd-CHIKV-E1 group (both *p* < 0.0001) (Figure 2b).

### 3.3. CHIKV Recombinant Adenovirus Vaccines Balances Th1/Th2 Responses

**Figure 3:**
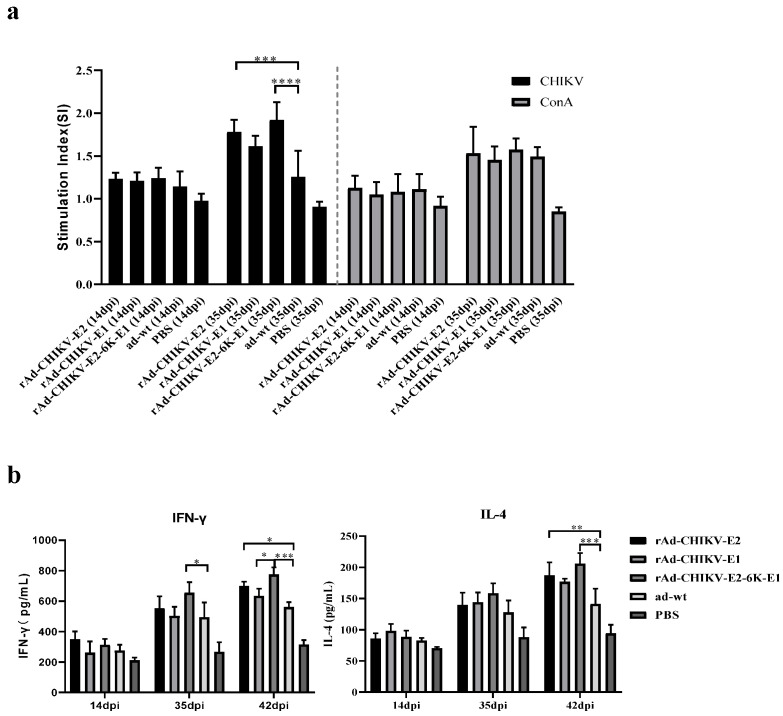
Analysis of cell mediated immune responses of mice immunized with recombinant adenovirus viruses. (**a**) Analysis of T-lymphocyte proliferative response in mice. The proliferative capacity of T-lymphocytes of each group under the stimulation of CHIKV antigen as specific stimulator and Con A as nonspecific stimulator. (**b**) Measure the level of IFN-γ and IL-4 of mice serum (n = 3) at 14 dpi, 35 dpi and 42 dpi (7 dpc) in each group. The cytokines were measured by a commercial mouse uncoated ELISA kit. Data represent the mean ± SD of each group. * *p* < 0.05, ** *p* < 0.01, *** *p* < 0.001, **** *p* < 0.0001.

Splenocytes of mice immunized with recombinant adenovirus vaccines showed enhanced proliferation when nonspecifically stimulated with ConA. After stimulation with inactivated CHIKV at 14 dpi, the proliferation levels of splenocytes of the rAd-CHIKV-E2-6K-E1 group were higher than those of the other two recombinant adenovirus vaccines groups with no significant difference. Especially, obviously proliferation levels in splenocytes of the rAd-CHIKV-E2-6K-E1 group were observed (Figure 3a).

The cytokines of all the immunization groups exhibited a balanced response of pro- and anti-inflammatory cytokines and showed an increase dependent on time. As the representatives of Th1 and Th2 cytokines, the levels of IFN-γ and IL-4 in three recombinant vaccine groups were all upregulated, which were higher than those of the ad-wt control group. Notably, at 14 dpi, there was no obvious difference between the immunization groups and the ad-wt control group. (Figure 3b). The levels of IFN-γ and IL-4 in mice immunized with rAd-CHIKV-E2-6K-E1 were 1.37 and 1.20 times higher than those immunized with the ad-wt at 35 dpi, respectively. Furthermore, the serum of mice in all experimental groups was collected for continuous cytokines monitoring, and the results showed that the levels of IFN-γ and IL-4 in rAd-CHIKV-E2-6K-E1 group were 1.45 and 1.38 times higher than those in ad-wt control group at 42 dpi, respectively (*p* < 0.001). The results showed that the recombinant adenovirus vaccines could balance Th1/Th2 response and were capable of producing adjuvant effects.

### 3.4. Protective Efficacy of Recombinant Adenovirus Vaccines against Viral Challenge In Vivo

**Figure 4:**
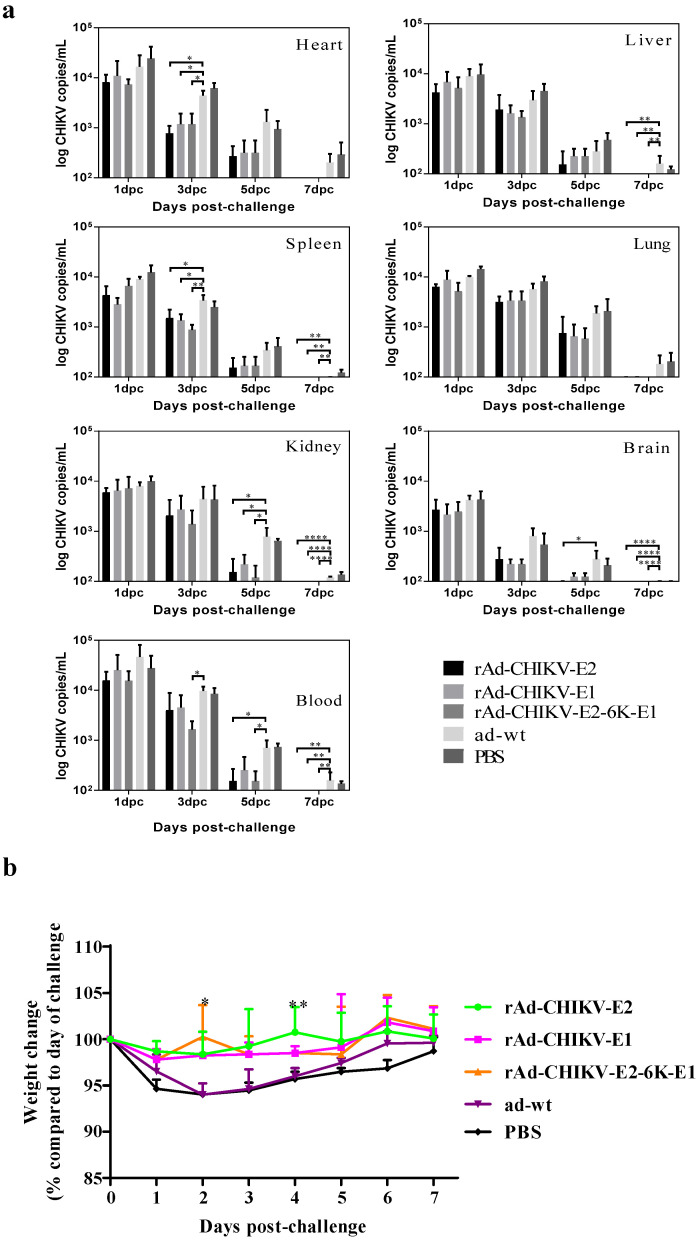
Immunized with recombinant adenovirus vaccines of CHIKV prevents the viremia and the weight loss in BALB/c mice. (**a**) The viral load of brain, heart, liver, spleen, lung, kidney, and blood of mice (n = 3) following challenge with CHIKV at 0, 1, 3, 5, and 7 dpc was measured by the quantitative RT-PCR. (**b**) The weight of mice (n = 4) following challenge with CHIKV were measured for 7 dpc. Data represent the mean ± SD of each group. * *p* < 0.05, ** *p* < 0.01, **** *p* < 0.0001.

Low levels of viremia were detected two weeks after the booster immunization. The viral loads of tissue samples in each immunization group were lower than in the control group. Especially, the heart, liver, kidney, brain, and blood tissues of mice significantly decreased after 3 dpc, while the lung and blood significantly decreased after 5 dpc. Besides, rAd-CHIKV-E2-6K-E1 and rAd-CHIKV-E2 immunized mice were able to inhibit viremia, but rAd-CHIKV-E2-6K-E1 did not show better inhibition ability of viremia and organ viral loads than rAd-CHIKV-E2. Low viral loads were detected in the heart, kidney, and blood of mice immunized with rAd-CHIKV-E2-6K-E1 and rAd-CHIKV-E2 groups decreased by 0.4–0.7 orders of magnitude compared with the ad-wt control group (Figure 4a). Moreover, none of the immunized mice suffered from diseases or died, or weights dropped significantly. In contrast, mice in the control group gradually lost 5% of their body weight at 1 or 2 dpc (Figure 4b). Taken together, the recombinant adenoviruses provided protection after the CHIKV challenge.

## 4. Discussion

At present, the commercial chikungunya vaccine is not available clinically. And multiple delivery approaches, including live-attenuated, inactivated, virus vector, DNA, and virus-like particle (VLP)-based candidate vaccines have also been tested and shown to be effective in providing protection against CHIKV [16,17,18,19]. Especially, the inactivated and VLP candidate vaccines are currently in Phase I clinical trials, and a live-attenuated vaccine is in Phase II clinical trials [16,20]. And there is still no approved vaccine or specific treatment for CHIKV infection. In this study, we constructed recombinant adenovirus vaccines expressing CHIKV E2/E1 and E2-E1 proteins, respectively. E2 and E1 proteins are the two important surface proteins that harbor the major viral epitopes and participate in the attachment and the entry of the virus into target cells [21]. Moreover, E2 and E1 proteins are the key targets of the host humoral immune responses and most anti-CHIKV vaccines [22,23].

Neutralizing antibodies are the major factors in providing protection against CHIKV [24]. Studies have found that the E2 produced in combination with E1 can induce higher neutralizing antibody responses than either protein alone [14]. Therefore, a recombinant adenovirus vaccine of co-expressing E2 and E1 was constructed to detect whether it could induce strong neutralizing antibody responses. Here, the recombinant adenovirus vaccine immunization groups were found to induce higher levels of anti-CHIKV IgG-specific neutralizing antibodies. However, there was no obvious difference in neutralizing antibodies with the same lower levels (1:256) between co-expressing E2-E1 and E2 expressing alone group. The presumed reason for these findings may be less immunogenic compared with the MVA vaccine which was used the complete structure of C-E3-E2-6K-E1 as an antigen. Similar applications were used to develop three preclinical CHIKV vaccines which used E3-E2-E1 or C-E3-E2-6K-E1 as antigens, and the expression of E2-E1 or E2 or E1 alone was found to be less immunogenic than the combination ones [25,26,27]. The vaccine immunization schedule was so short that antibodies did not reach peak levels, which might be the second reason for inducing lower humoral immune responses in mice.

Although T cells cannot prevent CHIKV infection, they are essential for clearing infected cells (including helper T cells) to form functional and long-term immunity [28]. Cell mediated immune responses against recombinant adenoviruses were evaluated by splenocyte proliferation and cytokine profiling. In this study, we isolated lymphocytes from mouse spleens and performed lymphocyte proliferation transformation experiments. The levels of lymphocyte proliferation in all the vaccine groups were significantly improved following stimulation with a CHIKV virus-specific antigen. Cytokines was detected from serum samples at 14 and 35 dpi. Th1 subsets secrete IL-2, IFN-γ, and other cytokines, which are responsible for resistance to most pathogens, whereas Th2 subsets secrete IL-4, IL-10, and other cytokines [29]. Recombinant replication-incompetent adenoviruses have an acceptable safety profile in humans and can induce a Th1-biased immune response in animals and humans, which are consistent with our experimental results [30,31]. The levels of IFN-γ and IL-4 were increasing significantly in both the immunization groups and the ad-wt control group. This may be due to the differential immunogens, vectors, or other reasons affecting Th1 and Th2 activation [10,28].

In the absence of an appropriate mouse or a small laboratory animal model for CHIKV infection, it had been difficult to evaluate the challenge. For CHIKV, researchers have infected adult BALB/c and C57LB mice, though no clinical symptoms were observed, temporal replication of the virus in the target organ(muscles)/brain/spleen and blood and seroconversion to anti-CHIKV antibodies was observed in both species [32]. In this study, no BALB/c mice died following the challenge with CHIKV. Besides, rAd-CHIKV-E2-6K-E1 did not show better anti-CHIKV effects than rAd-CHIKV-E2, but better than rAd-CHIKV-E1. The reason may be that the viral pathogenesis was too weak, or the mouse model was not the most suitable for CHIKV. Compared with the ad-wt control group, the immunized mice lost weight after the CHIKV challenge, and the viral load in organs and blood decreased significantly. This finding indicates that the recombinant adenovirus vaccines can inhibit CHIKV replication in mice.

## 5. Conclusions

In conclusion, we describe the development, identification, and immunization efficacy of recombinant adenovirus vaccines based on the envelope protein of E2/E1/E2-6K-E1 of CHIKV. The recombinant adenovirus vaccine candidates could induce high levels of neutralizing antibodies and significantly reduce the viral loads after challenging CHIKV. However, compared with rAd-CHIKV-E2, the rAd-CHIKV-E2-6K-E1 vaccine did not significantly induce better immune effects. Overall, these data suggest that the recombinant adenovirus is a potential candidate vaccine against CHIKV.

## Authors Contributions

H.Z., H.L. and N.J. designed the experiments. L.C., W.W., W.S., J.Z. and Z.H. performed the animal experiments. L.C., H.Z., C.X., Y.X. and J.H. collected and analyzed the data. L.C. and H.Z. wrote the manuscript. H.L. and N.J. reviewed the manuscript. All authors have read and agreed to the published version of the manuscript.

## Figures and Tables

**Table 1:** Grouping details of immunization and challenge test of mice.

Group	Injection	Injection Route	Injection Dose	Challenge	Challenge Route	Challenge Dose
I	rAd-CHIKV-E2	i.m.	10^9^ pfu/100 μL	CHIKV	i.v.	10^4^ pfu/100 μL
II	rAd-CHIKV-E1	i.m.	10^9^ pfu/100 μL	CHIKV	i.v.	10^4^ pfu/100 μL
III	rAd-CHIKV-E2-6K-E1	i.m.	10^9^ pfu/100 μL	CHIKV	i.v.	10^4^ pfu/100 μL
IV	ad-wt	i.m.	10^9^ pfu/100 μL	CHIKV	i.v.	10^4^ pfu/100 μL
V	PBS	i.m.	100 μL	CHIKV	i.v.	10^4^ pfu/100 μL
